# Point mutation in the stop codon of *MAV_RS14660* increases the growth rate of *Mycobacterium avium* subspecies *hominissuis*

**DOI:** 10.1099/mic.0.001007

**Published:** 2020-12-23

**Authors:** Tomomi Kawakita, Tetsu Mukai, Mitsunori Yoshida, Hiroyuki Yamada, Masaaki Nakayama, Yuji Miyamoto, Masato Suzuki, Noboru Nakata, Takemasa Takii, Akihide Ryo, Naoya Ohara, Manabu Ato

**Affiliations:** ^1^​ Department of Mycobacteriology, Leprosy Research Center, National Institute of Infectious Diseases, Tokyo, Japan; ^2^​ Department of Microbiology and Molecular Biodefense Research, Yokohama City University Graduate School of Medicine, Yokohama, Japan; ^3^​ Department of Mycobacterium Reference and Research, Research Institute of Tuberculosis, Tokyo, Japan; ^4^​ Department of Oral Microbiology, Okayama University Graduate School of Medicine, Dentistry and Pharmaceutical Sciences, Okayama, Japan; ^5^​ Advanced Research Center for Oral and Craniofacial Sciences, Okayama University Dental School, Okayama, Japan; ^6^​ Antimicrobial Resistance Research Center, National Institute of Infectious Diseases, Tokyo, Japan

**Keywords:** *Mycobacterium avium *subspecies *hominissuis*, single nucleotide replacement, growth rate, sliding motility

## Abstract

*
Mycobacterium avium* subspecies *hominissuis* (MAH) is a pathogen that causes various non-tuberculous mycobacterial diseases in humans and animals worldwide. Among the genus, MAH is characterized by relatively slow growth. Here, we isolated a rapidly growing variant of the MAH 104 strain. The variant strain (named N104) exhibited an enhanced growth rate and higher motility compared to the parent MAH 104 strain (P104). Whole-genome sequencing analysis of N104 revealed the loss of the stop codon of *MAV_RS14660* due to a single nucleotide replacement, resulting in the substitution of the codon for tryptophan. Notably, exclusion of the stop codon ligated the open reading frames and caused the fusion of two adjacent proteins. A revertant parent strain, in which a mutation was introduced to restore the stop codon, revealed that elimination of the stop codon in *MAV_RS14660* was responsible for the N104 phenotype. Furthermore, we analysed the phenotypes of the parent and mutated strains by determining the functions of the *MAV_RS14660* and *MAV_RS14655* coding regions flanking the stop codon. The mutant strains, expected to express a fusion protein, exhibited increased resistance to antimicrobial drugs and exogenous copper toxicity compared to that of the parent strains. These findings suggest that the fusion of the *MAV_RS14660*- and *MAV_RS14655*-encoding regions in the mutant N104 strain could be related to the modified functions of these intrinsic proteins.

## Introduction

*Mycobacterium* spp. grows more slowly than other bacterial species. The doubling time of *Mycobacterium* spp. varies from 4 h to 12 days [1, 2]. Runyon proposed doubling time as a parameter for the classification of *Mycobacterium* in 1959 [3]. The colonies of slow-growing mycobacteria generally require more than 7 days to grow to a detectable size on solid medium [4]. Slow growth is an important strategy for *Mycobacterium* to escape from attack by the host immune system and establish persistent infection for decades in the host. However, the mechanisms involved in the slow growth of mycobacteria are not yet fully understood. One of the factors involved in determining the growth of mycobacteria is metabolic activity. rRNA synthesis [5], regulators of stress response sigma factors and a resuscitation-promoting factor [6] are involved in regulating the rate of growth of *Mycobacterium *. Specific mutations in *rpoB*, which encodes an RNA polymerase subunit, have been reported to reduce growth [7]. Moreover, ATPases [8] and serine/threonine kinases [9] regulate the growth rate of *Mycobacterium *.

Components of the cell wall also exert a significant effect on mycobacterial growth rate. The two-component MtrAB system has been implicated in cell division and maintenance of cell wall integrity [10]. A deletion mutant of *rpoZ*, encoding the ω subunit of RNA polymerase, exhibits slow growth rate and reduced sliding motility, which is the ability to spread on the surface of a solid medium in a flagellum-independent manner [11]. Enhanced sliding motility in mycobacteria helps detect colonies on solid agar early, which may be regarded as increased growth rate. Glycopeptidolipids (GPLs), expressed on the outer membrane of *Mycobacterium smegmatis* and *Mycobacterium avium *, are essential factors that induce sliding motility [12–14].

*M. avium* is a slow-growing mycobacterial species that consists of three subspecies: *M. avium* subsp. *avium *, *M. avium* subsp. *paratuberculosis* and *M. avium* subsp. *hominissuis* (MAH). MAH is clinically important because it is the causative pathogen of various infectious diseases in humans and animals worldwide. The MAH 104 strain was isolated from an adult patient with AIDS in 1983 and is used as a reference genome strain.

We isolated a rapidly growing variant of MAH 104 strain. The colonies of the parent MAH 104 strain took more than 6 days to grow to a detectable size on solid medium, whereas the variant, obtained during routine culture by coincidence formed detectable colonies within 5 days. This finding led us to speculate regarding the presence of a genetic modification responsible for variation in growth rate and/or sliding motility in the variant strain. Therefore, we analysed the phenotypes of the variant strain and performed comparative genome analysis using the parent MAH 104 strain.

## Methods

### Bacterial strains and growth conditions

The MAH 104 strain (represented as P104 in this study) was provided by Dr William Bishai, Johns Hopkins School of Medicine, Baltimore. All the MAH strains were grown in Middlebrook 7H9 broth supplemented with albumin–dextrose–catalase (ADC; Difco BD, Franklin Lakes, NJ, USA) and 0.05 % (v/v) Tween 80 (7H9 broth) or on Middlebrook 7H10 agar supplemented with oleic acid–albumin–dextrose–catalase (OADC; Difco) and 0.5 % (v/v) glycerol (7H10 agar plate) at 37 °C. The *M. smegmatis* mc^2^ 155 strain, used for the production of recombinant proteins, was grown in Luria–Bertani (LB) broth supplemented with 0.05 % (v/v) Tween 80 at 37 °C. *Escherichia coli* DH5α, used for cloning, was grown in LB broth at 37 °C. The following antibiotics were used to screen the transformants: ampicillin (100 µg ml^−1^), kanamycin (50 µg ml^−1^) and hygromycin (100 µg ml^−1^).

### Bacterial growth and colony-forming units (c.f.u.)

Initial bacterial suspension was adjusted to 0.04–0.05 OD_530_, and 10 ml of the suspension was incubated at 37 °C with shaking. Bacteria were cultured for 10 days and growth was monitored by measuring the OD_530_ once a day. c.f.u. were enumerated once a day for 3 days. Generation time was calculated according to the following formula reported by Elguezabal *et. al* [15]: *G*=ln2 (*t*_f_–*t*_0_) (ln*N*_f_−ln*N*_0_)^−1^, where *N*_f_: is the number of bacteria at the final time point, *N*_0_is the number of bacteria at the initial time point, *t*_f_ is the final time point, *t*_0_ is the initial time point and *G* is the generation time. We set the final time point at 3 days of culture. Data obtained from four independent experiments are represented as the mean±standard deviation.

### Colony morphology

Colony morphology was observed using a light microscope (CKX41, OLYMPUS, Tokyo, Japan) after 5, 8 and 14 days of culture on a 7H10 agar plate. The diameter of the colonies (major axis) was measured from images.

### Sliding motility assay

Bacterial suspensions grown on 7H10 agar were inoculated on a 0.3 % (w/v) agar plate containing 7H9 medium supplemented with ADC and 0.5 % (v/v) glycerol. After incubation at 37 °C for 5 days, the diameter of the colonies was measured.

### Electron microscopy and measurement of bacterial size

Morphological analysis of bacterial cells using scanning electron microscopy (SEM; IT-300, JEOL, Tokyo, Japan) was performed as described previously [16]. Briefly, colonies that had been cultured on 7H10 agar for more than 20 days were fixed with 2.5 % (w/v) glutaraldehyde in 0.1 M phosphate buffer (PB; pH 7.4) at 4 °C overnight and rinsed thrice with PB. Next, the samples were fixed with 1 % (w/v) osmium tetroxide for 1 h at 4 °C and dehydrated using graded series of ethanol. After substitution with *t*-butyl alcohol, the samples were dried using a freeze-drying device (JFD-320, JEOL, Tokyo, Japan) and coated with gold using an ion spatter coater (Q150 ES Plus, Quorum Technologies, Laughton, UK). The samples were observed using SEM. The long axis of bacteria was measured from the acquired electron micrographs. Morphological analysis of bacterial cells using cryo-transmission electron microscopy (Cryo-TEM) was performed as described previously [17]. Briefly, 7H9 bacterial cultures were grown to ~0.5 OD_530_ under conditions of shaking and were fixed with 2.5 % (w/v) glutaraldehyde in PB at 4 °C overnight. Then, after centrifugation at 10 000 ***g***, the supernatants were discarded and the pellet was rinsed with PB. Finally, the pellets were resuspended in 200 µL of PB from which 1 µl of suspension was applied to a glow-discharged carbon grid with holes (Quantifoil copper grids R 2/1 or S 7/2, Quantifoil MicroTools, Jena, Germany) and mounted in an environmentally controlled chamber at 100 % humidity. The grids were frozen in vitreous ice by plunging them into a liquid ethane–propane mixture cooled with liquid nitrogen using EM GP2 (Leica Mikrosysteme GmbH, Vienna, Austria). The grid was loaded in Single Tilt Liquid Nitrogen Cryo Transfer Holder (model 626, Gatan, Inc., Pleasanton, CA, USA) and subjected to TEM (JEM-1230, JEOL, Tokyo, Japan). The microscope was operated at 120 kV acceleration voltage and during examination the samples on the grids were cooled with liquid nitrogen to a temperature of −175.9 to −177.8 °C. Raw images of the intact cells were recorded at magnifications of ×10 000,×15 000, or ×20 000 according to the length of a single cell using the 1×1K CCD digital camera system (OSIS MegaView G2, Olympus, Tokyo, Japan). The images captured with Cryo-TEM were analysed using the Measure command in the Analyse menu of ImageJ/Fiji [18].

### Preparation of DNA and whole-genome sequence analysis

Genomic DNA was extracted using a modified previously described method [19]. The bacterial cells were concentrated by centrifugation, and the pellet was resuspended in 50 mM Tris/HCl (pH 8.0), 10 mM EDTA, treated with lysozyme (Sigma-Aldrich, St Louis, MI, USA) and incubated at 37 °C overnight. Subsequently, sodium dodecyl sulphate (SDS) and proteinase K were added to the samples at final concentrations of 10 and 1 mg ml^−1^, respectively, and the samples were incubated at 50 °C for more than 1 h. Subsequently, DNA was purified using a standard phenol/chloroform method.

Genomic DNA of each strain was used to generate the Nextera XT library for genome sequencing in accordance with Illumina MiniSeq 2×300 bp paired-end protocol.

To detect variants between the P104 and N104 strains, single-nucleotide polymorphisms (SNPs) were analysed as described previously with some modifications [20]. In brief, raw sequence reads of P104 and N104 strains were mapped against publicly available complete genome sequence of MAH 104 (accession no. NC_008595) using the BWA program [21]. After improving the alignments using the ‘RealignerTargetCreator’ and ‘IndelRealigner’ functions of GATK, the ‘HaplotypeCaller’ function with the ‘-ploidy 1’ option was used to detect SNPs and short indels [22, 23]. The ‘VariantFiltration,’ ‘BaseRecalibrator’ and ‘PrintReads’ functions of GATK were used to filter false positives, and the remaining SNPs and short indels were annotated with SnpEff [24]. To validate the detected SNPs, we performed whole-genome alignment between the P104 and N104 strains. The Illumina reads of P104 and N104 strains were *de novo*-assembled using the Shovill pipeline with default settings (https://github.com/tseemann/shovill). The assembled genomes of P104 and N104 strains were annotated using DFAST [25] and used for whole-genome alignment performed using progressiveMauve [26].

### RNA preparation

MAH strains were grown at 37 °C to an OD_530_ of ~0.5. Cells were collected by centrifugation at 2000 ***g*** for 10 min and washed twice with phosphate-buffered saline (PBS). The cell pellets were resuspended in 1 ml of RNApro solution (MP Biomedicals, Solon, OH, USA) and immediately transferred to a 2 ml screw cap tube containing 0.1 mm diameter glass beads and homogenized using Micro Smash MS-100 (TOMY Digital Biology, Tokyo, Japan) at 4500 r.p.m. thrice for 45 s. RNA was extracted according to the manufacturer’s protocol. Residual genomic DNA was removed by DNase (Nippon Gene, Toyama, Japan) treatment, and the RNA was cleaned up using the RNeasy Mini kit (Qiagen, Hilden, Germany). The quality and quantity of RNA were analysed using NanoDrop (Thermo Fisher, Wilmington, DE, USA).

### Reverse transcription polymerase chain reaction (RT-PCR)

For conventional PCR, RNA was reverse-transcribed using the SuperScriptIV VILO Master Mix (Invitrogen Thermo Fisher Scientific, MA, USA) according to the manufacturer’s protocol. PCR was performed using primers MAV_RS14660 F1 and MAV_RS14655 R1. The PCR mixture consisted of 0.02 U KOD-Plus Neo (Toyobo, Tokyo, Japan), 10× PCR buffer, 0.2 mM dNTPs, 1.5 mM MgSO_4_, 0.2 µM of each primer and 1 µl of cDNA template. PCR was performed under the following conditions: initial denaturation for 2 min at 94 °C followed by 30 cycles of denaturation for 2 min at 98 °C, annealing for 30 s at 55 °C and an extension for 1 min at 60 °C. PCR products were analysed using 2 % (w/v) agarose gel electrophoresis.

For quantitative analysis, cDNA was prepared using the ReverTra Ace qPCR RT Master Mix with gDNA Remover (Toyobo) according to the manufacturer’s protocol. Template RNA was adjusted to 400 µg 10 µl^−1^ of mixture. Quantitative PCR was performed using the THUNDERBIRD Next SYBR qPCR Mix (Toyobo) and primers MAV_RS14660 F2, MAV_RS14660 R1, MAV_RS14655 F2 and MAV_RS14655 R2. We used 16S rRNA levels as the normalizing control. We used 1 µl of the cDNA templates. PCR was performed using Applied Biosystems StepOnePlus (Thermo Fisher) under the following conditions: predenaturation at 95 °C for 30 s, 40 cycles of denaturation at 95 °C for 5 s and annealing at 60 °C for 30 s followed by melting curve acquisition.

### His-tag staining of recombinant proteins

The mycobacterial expression vector, p2HPacC, was constructed on the basis of a previous report [27] with some modification, replacing the cloning site to cloning using the InFusion system (Takara, Shiga, Japan). MAV_RS14660, MAV_RS14655 and MAV_RS14660-MAV_RS14655 fusion open reading flames (ORFs) were amplified from N104 genomic DNA with the primer pairs, F15Pac MAC 1/R 15C6 MAC 1179, F15 Pac MAC 1183/R15 C6 MAC 2133 and F15Pac MAC 1/R15 C6 MAC 2133, respectively (Table 1). PCR products were cloned into p2HPacC vector with the InFusion system and verified with sequencing. C-terminal 6His-tagged recombinant proteins were expressed in *M. smegmatis* mc^2^ 155 driven by acetamidase promoter. Proteins were separated using sodium dodecyl sulfate/polyacrylamide gel electrophoresis followed by staining using the InVision His-tag In-gel Stain kit (Thermo Fisher Scientific, MA, USA).

**Table 1.** Oligonucleotide sequences of primers used in this study

Primer name	Sequence (5′−3′)	Comment
**Cloning primers for plasmid containing allelic exchange substrate**		
F UP MAC 1012* HindIII	TATAAAGCTTACGCTGTACCGCAATCACAT	Upstream F
R UP MAC 2277 XbaI	TTAATCTAGATTTGCGGGTGCTTTCGCACA	Upstream R
F DO MAC 2002 BamHI	TATAGGATCCAGCACCGTCGACAATGTCGA	Downstream F
R DO MAC 2717 KpnI	ATTAGGTACCGATTTCGGTGATGACGACGT	Downstream R
**Sequencing primers**		
F MAC 904 seq	GCCAACATCGAGACGTTCTT	
F MAC 1432 seq	AAGTCGGTGGTGGTGTTTCG	
R MAC 2870 seq	CTGGGTGGGTTTGGTCTTGA	
**Primers for RNA analysis**		
MAV_RS14660-F1	AACAACGGAGTGACGCTGAT	
MAV_RS14655-R1	CGCCCTGGTAGGTGATGAAG	
MAV_RS14660-F2	CGCTCAAGCCGTTCATCAAG	
MAV_RS14660-R2	GGGATCGCCAACGATGATCT	
MAV_RS14655-F2	GGGCTCAACGACCAGAAGAA	
MAV_RS14655-R2	GAAACACCACCACCGACTTG	
16S rRNA-F	AATTCCTGGTGTAGCGGTGG	
16S rRNA-R	GTTTACGGCGTGGACTACCA	
**Primers for recombinant proteins**		
F15Pac MAC 1	AGAAAGGGAGTCCACATGAAGATGTCAGGCCTGCT	
R 15C6 MAC 1179	GTGATGGTGGTGATGGGTGACCCACTTCTGCACC	
F15 Pac MAC 1183	AGAAAGGGAGTCCACATGATCTCGCTACGCCAACA	
R15 C6 MAC 2133	GTGATGGTGGTGATGCTGAGCGACCGTGATCGA	

*MAV_RS14660 start codon is numbered as 1.

### Preparation of recombinant MAH

The recombinant MAH strains in which the MAH N104 type gene was replaced with the MAH P104 type gene or the original MAH N104 type gene (as control) were established using a modified recombination protocol [28]. MAH N104 was cultured in Middlebrook 7H9 broth containing OAD, 0.2 % (v/v) glycerol and 0.05 % (v/v) tyloxapol or Middlebrook 7H10 agar supplemented with OADC and 0.2 % (v/v) glycerol. pJV53, encoding a recombinase, was transformed into electrocompetent N104 cells, wherein the expression of recombinase was induced by 0.2 % (w/v) acetamide. Subsequently, the induced N104 cells were electroporated. Allelic exchange substrates in the plasmid were amplified using PCR and F UP MAC1012 HindIII/R DO MAC2717 KpnI followed by transformation into the recombinase-expressing cells. After plating on 7H10 agar containing 50 g ml^−1^ hygromycin, the transformed cells were incubated to select transformants. The colonies of the transformed cells were screened using colony PCR based on the F MAC 904 seq/R MAC 2870 seq designed to amplify the whole exchanged sequence from outside the manipulated region. Homologous recombination was confirmed via sequencing using the primers listed in Table 1. The selected colonies were incubated in 7H9 broth for 1 week and plated on 7H10 agar without antibiotics. After the selection of pJV53-free rMAH, the cells were electroporated. pYUB870, which encodes a res site-specific resolvase, was introduced into rMAVs to excise the res sites flanking the *hyg* cassette. After selection on 7H10 agar containing 20 mg ml^−1^ kanamycin, the absence of the *hyg* cassette was confirmed using PCR with the F MAC 1432/R MAC 2870 primer pair. The recombinants were cultured in 7H9 broth without antibiotics, and the selected pYUB870-free rHAH were amplified using PCR based on F MAC 904 seq/R MAC 2870 seq; we confirmed the sequence and excision of the *hyg* cassette. The recombinant strain containing wild-type or mutated sequence was named NP or NN, respectively.

### Construction of plasmids containing allelic exchange substrates

Plasmids containing allelic exchange substrates that allow homologous recombination between plasmid-containing *MAV_RS14660–MAV_RS1655* and corresponding chromosomal regions in MAH N104 were constructed following a previously described method [28, 29]. The genomic DNAs were extracted from MAH P104 or MAH N104. An approximately 1.3 kb long fragment containing the 3′ region of *MAV_RS14660* and complete *MAV_RS1655* was amplified using F UP MAV 1012 HindIII /R UP MAC 2277 XbaI (Table 1). Similarly, a 0.7 kb long fragment containing MAV_RS14650, a gene located downstream of MAV_RS14655, was amplified using F DO MAC 2002 BamHI / R DO MAC 2717 KpnI (Table 1). The PCR product of the former was digested with HindIII and XbaI and cloned upstream of the hygromycin resistance cassette (*hyg*) in pΔAHm31 (Table 2). The PCR product of the latter was digested with BamHI and KpnI and cloned downstream of *hyg*. pΔAHmP contained the wild-type stop codon (TAG), while pΔAHm N contained the mutated sequence TGG. The cloned nucleotide sequences of the plasmids were verified using Sanger sequencing.

**Table 2.** Plasmids used in this study

Name	Characteristics	Reference
pΔAHm31	hyg cassette flanked with res sites in pUC18; excludes Ampr sequence, Hyg^r^	[28]
pΔAHm P	MAV_14660 stop codon is wild-type (TAG)	This study
pΔAHm N	MAV_14660 stop codon is mutated (TGG)	This study
pJV53	Shuttle plasmid expressing recombinase under the control of acetamide, Kan^r^	[29]
pYUB870	Shuttle plasmid expressing resolvase, Kan^r^	[45]
p2HPacC	MAV_RS14660, MAV_RS14655 and MAV_RS14660-MAV_RS14655 fusion ORFs with C-terminal 6His tag, Hyg^r^	This study

### Antimicrobial susceptibility test

Antimicrobial susceptibility was determined using 7H9 broth microdilution. A range of twofold dilution series of antimicrobial agents was prepared as follows: clarithromycin (0, 0.02–16 µg ml^−1^), rifampicin (0, 0.004–4 µg ml^−1^), ethambutol (0, 0.06–64 µg ml^−1^) and levofloxacin (0, 0.01–8 µg ml^−1^). Each concentration agent was aliquoted into 96-well plates (100 µl). The concentration of bacterial suspension was adjusted to 0.2 OD_530_ and diluted 1 : 50 in 7H9 broth. The bacterial suspension was inoculated (10 µl) in each well. After incubation at 37 °C for 10 days, we determined the minimal inhibitory concentrations. The concentrations of antimicrobials that were added to 7H10 agar plates for susceptibility testing were as follows: clarithromycin (0.06 µg ml^−1^), rifampicin (0.06 µg ml^−1^), ethambutol (1 µg ml^−1^) and levofloxacin (0.5 µg ml^−1^). The concentration of bacterial suspension was adjusted to 0.2 OD_530_ and a series of 10-fold dilutions was prepared up to 10^−7^. The diluent was inoculated on 7H10 agar plates containing antimicrobials (2 µl/spot). The plates were incubated at 37 °C for 2 weeks.

### Congo red binding assay

The Congo red binding ratio was determined as described previously [30]. Briefly, the optical density of the bacterial culture grown in 7H9 broth was adjusted to 1.0 OD_530_. Then, the culture was supplemented with 100 µg ml^−1^ of Congo red and incubated for 2 days. Later, the bacterial cells were washed twice with PBS and resuspended in acetone with shaking for 2 h. After centrifugation, the supernatant was analysed spectrophotometrically at 488 nm. The Congo red binding rate was evaluated as the measured value divided by the optical density of the original bacterial suspension at OD_650_.

### Copper resistance test

Copper resistance was assessed following the method reported by Wolschendorf *et al*. with some modifications [31]. Briefly, the concentration of bacterial suspension was adjusted to 0.2 OD_530_ followed by preparation of a series of 10-fold dilutions up to 10^−7^. The diluent was inoculated on 7H10 agar plates (2 µl/spot) containing 25, 50 and 100 µM CuSO_4_. The plates were incubated at 37 °C for 2 weeks.

## Results

### Variant strain (N104) showed rapid growth compared to the parent strain (P104)

**Fig. 1.**
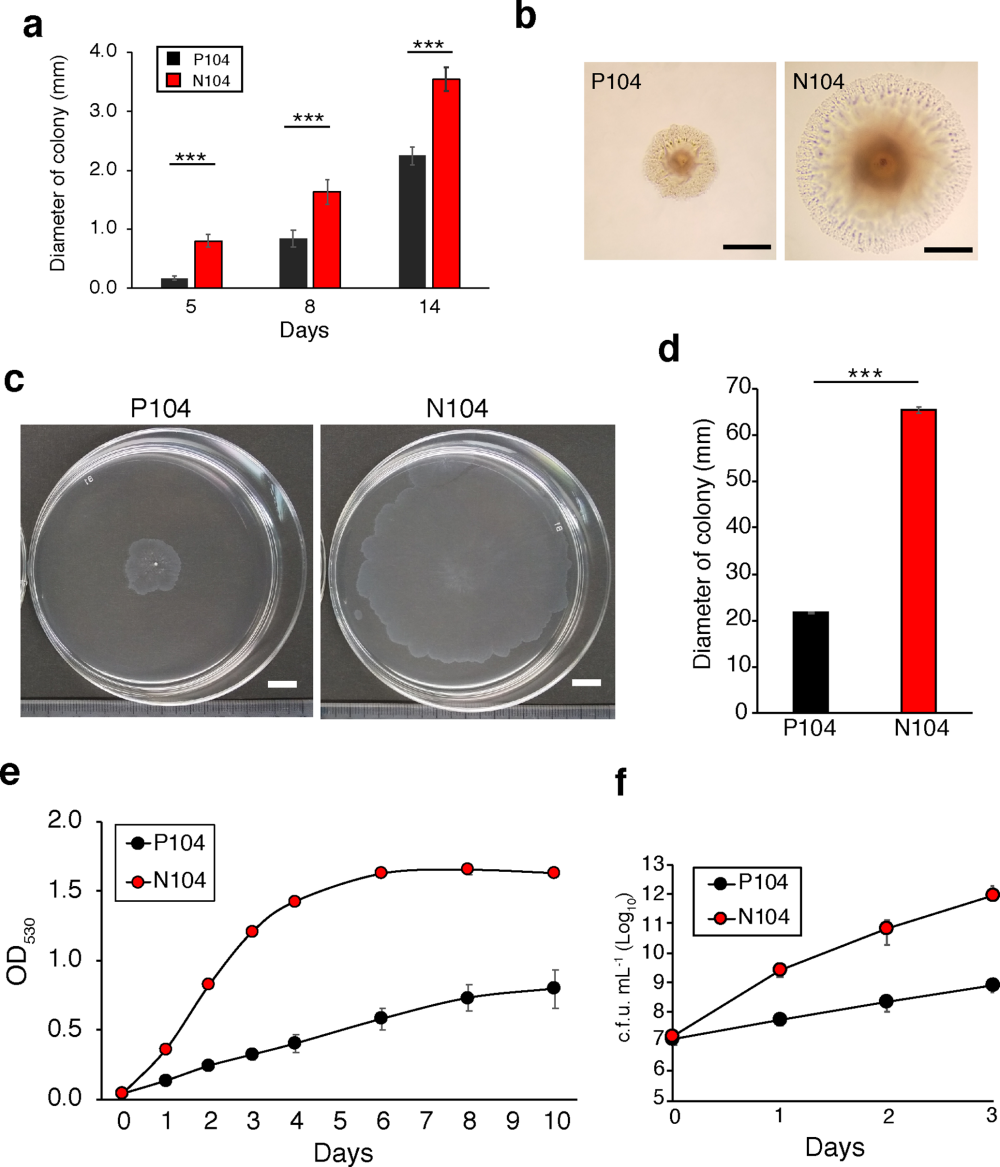
Phenotype of parent (P104) and variant (N104) MAH 104 strains. (a) Diameter of colonies of MAH 104 strains on agar plates were measured after *n* days of culturing, as indicated in the figure. Data are represented as he mean±standard deviation (sd) of 10 samples in each group. (b) Representative images of colonies of the two strains on 7H10 agar after 8 days of growth. Scale bars represent 0.5 mm. (c, d) Motility of the two strains on 0.3 % agar. (c) Colonies on 0.3 % agar after 5 days of growth. Scale bars represent 10 mm. (d) Diameter of colonies as measured after 5 days of culture. Data are represented as the mean±sd obtained from *n*=4. (e, f) Growth of MAH 104 strains in 7H9 broth under conditions of shaking at 37 °C, analysed at OD_530_ (e) and based on colony-forming units (c.f.u.) (f) after *n* days of culturing as indicated in the figure. Data are represented as the mean±sd obtained from (c) *n*=3 and (d) *n*=4. Student’s *t*-test was used for statistical analysis. ***P<0.005.

To investigate whether the difference in growth rate of the variant MAH 104 strain (N104) and parent MAH 104 strain (P104) was measurable, we determined the colony size of the two strains on 7H10 agar. Colonies of N104 were significantly larger than those of P104 during the entire period of culture (Fig. 1a). There was no difference in the morphology of colonies of N104 and P104 after 8 days of culturing (Fig. 1b). The diameter of the P104 colonies did not reach the diameter of N104 even after more than 20 days of culture. After this period, N104 colonies were raised with spreading edges, while those of P104 were raised without spreading edges (Fig. S1, available in the online version of this article). *M. avium* has the ability to spread on solid surfaces via its sliding motility, which was observed on semisolid medium [13, 14]. Therefore, we assessed the motility of two strains by measuring the colony size on 0.3 % agar plate. N104 colonies were spread wider than the P104 colonies after 5 days of culture (Fig. 1c, d). Next, to compare the growth rate of each strain, N104 and P104 strains were diluted to a starting optical density of 0.04–0.05 at 530 nm and were incubated in liquid medium at 37 °C with shaking, following which the OD_530_ was measured. The N104 strain grew faster than the P104 strain at all the time points, especially during the early exponential phase (Figs 1e and S2). To determine the initial growth of strains, we analysed the c.f.u. during the first 3 days of culture. The growth rate of N104 increased compared with that of P104 (Fig. 1f). The generation times, based on c.f.u., obtained from 3 day cultures were 12±2 h and 5±1 h for P104 and N104, respectively. These data indicated that N104 grew faster than P104 did. Thus, the early detection of N104 compared to that of P104 was caused by an increase in the growth rate and high spreading ability of N104 cells compared to that of P104 cells.

### N104 cells were shorter than P104 cells

**Fig. 2.**
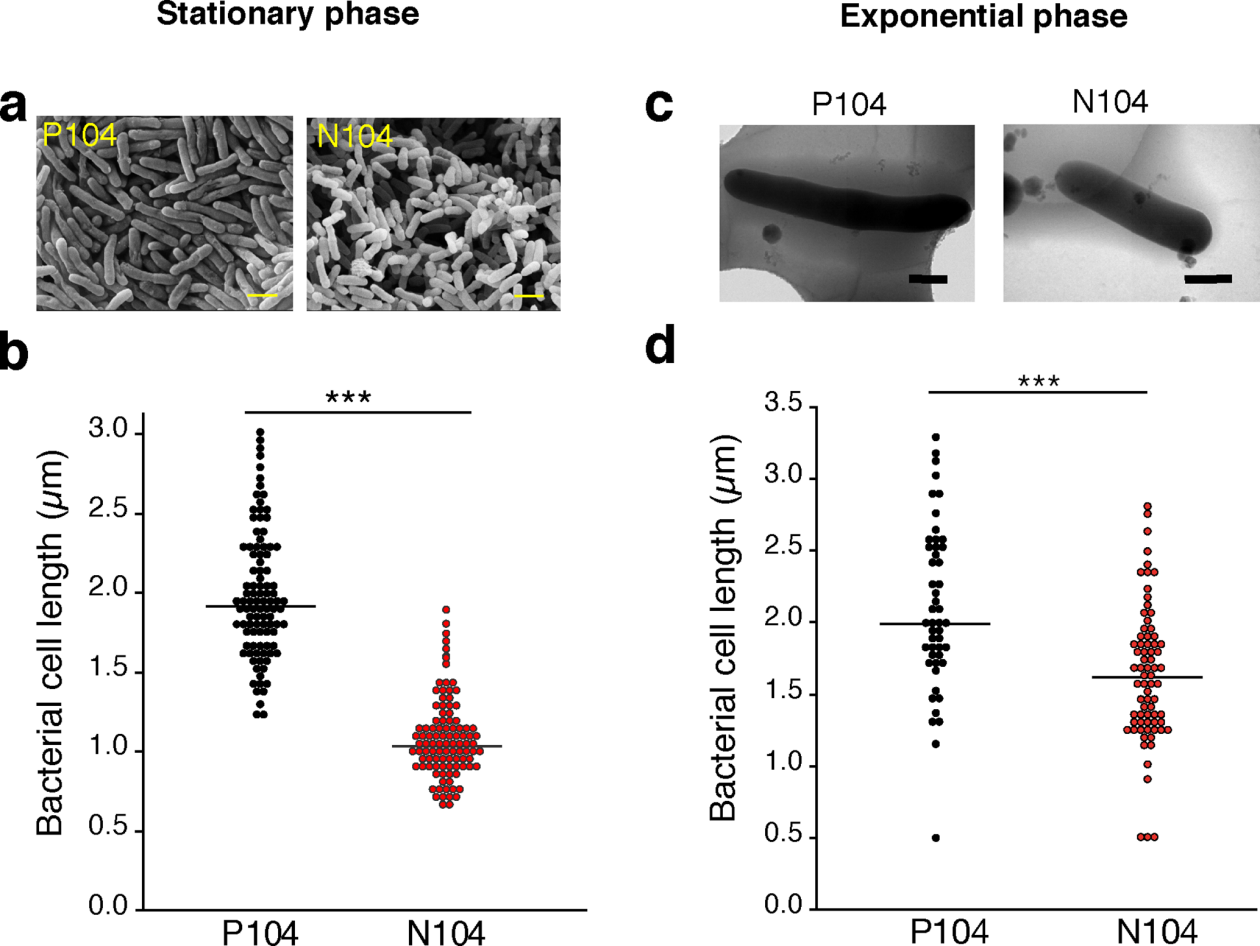
Bacterial morphology. (a, b) Bacterial cells on solid 7H10 medium after more than 20 days of culturing were observed using scanning electron microscopy. (a) Electron micrographs of P104 and N104 are shown. Scale bar represents 1 µm. (b) Length of bacterial cells measured from using the scanning electron micrographs. Bar indicates median (*n*=100). (c, d) Bacterial cells harvested from 7H9 liquid medium at 0.5 OD_530_ and observed using cryo-transmission electron microscopy (Cryo-TEM). (c) Representative micrographs of strains P104 and N104. Scale bar represents 0.5 µm. (d) Length of bacterial cells was measured using the Cryo-TEM (P104, closed circle; N104, open circle). Bar indicates median (P104, *n*=49; N104, *n*=73). he Mann–Whitney U test (b, d) was used for statistical analysis. ***P<0.005; ns, not significant.

To explore the differences in he micromorphology of P104 and N104 cells, we subjected the bacteria to SEM. Observation of colonies on agar plates in the stationary phase revealed that the cells of the N104 strain were significantly shorter than those of the P104 strain (Fig. 2a, b), with similar cell width (Fig. S3). Next, the length of the cells grown in liquid medium during exponential growth was determined using Cryo-TEM. N104 cells were significantly shorter than P104 cells (Fig. 2c, d). Taken together, N104 exhibits different micromorphologies than the P104 strain.

### N104 bore a nonsynonymous substitution that deleted the stop codon of an MAH protein

**Fig. 3.**
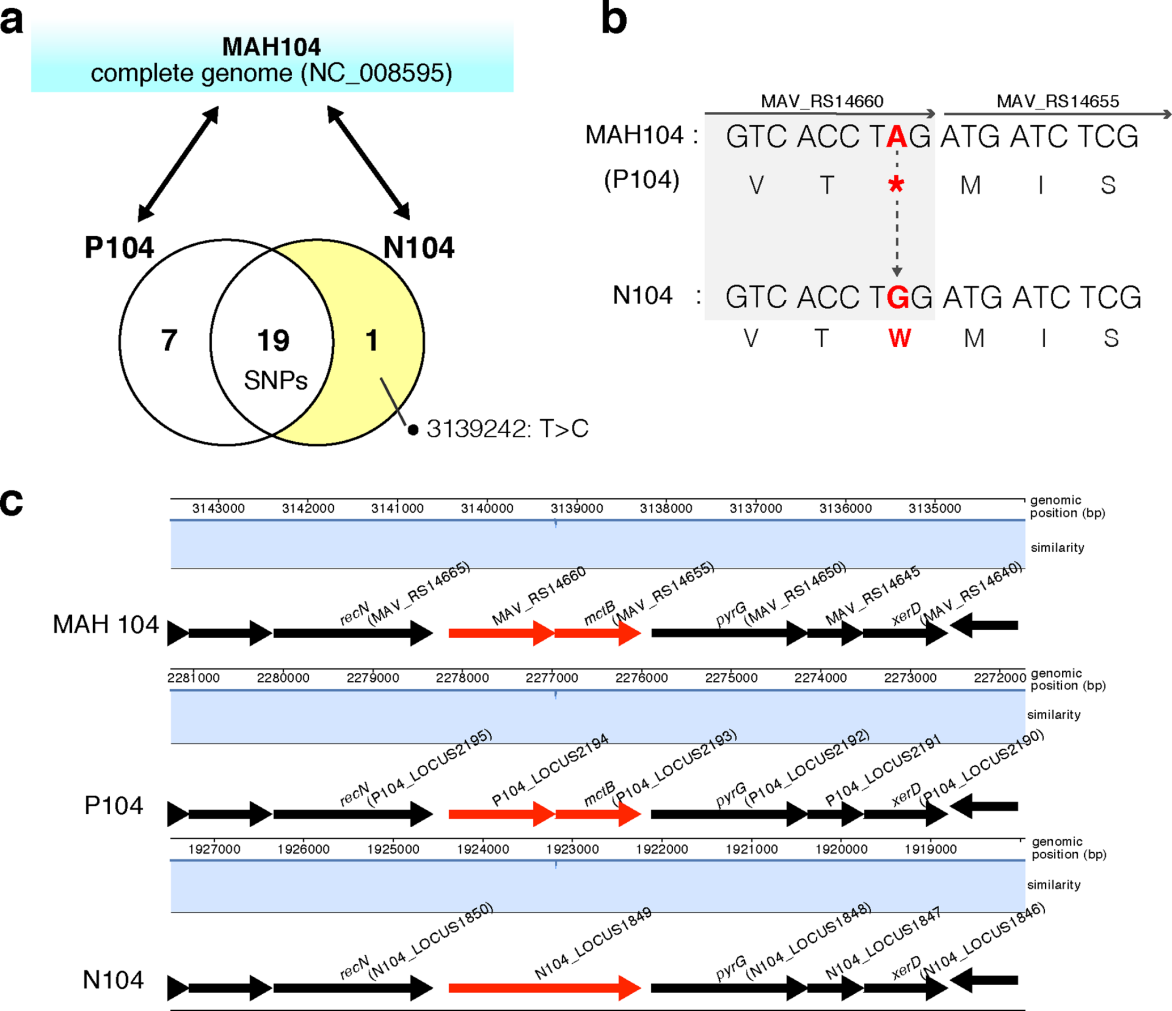
Comparative whole-genome analysis of the MAH 104 strains. (a) Schematic diagram of the strategy used for detecting the responsible mutation. Sequence reads were mapped to the MAH 104 reference sequence (NC_008595). Each digit represents the number of single-nucleotide polymorphisms (SNPs) detected as P104-specific, common between P104 and N104, and N104-specific upon comparison with the MAH 104 genome sequence. (b) The position of the N104-specific SNP in *MAV_RS14660*. The nucleotide and amino acid sequences of *MAV_RS14660* and *MAV_RS14655*. Bold letter represents N104-specific SNP (3139242: T>C). (c) A progressiveMauve alignment of MAH 104, P104 and N104. Each genome was laid out in a track and white boxes indicate annotated genes. A similarity plot is shown for each genome; the height of the plot corresponds to the sequence identity in that region.

To identify the genetic changes responsible for the phenotypes of the N104 strain, we compared the genomes of N104 and P104 using whole-genome sequencing. Upon mapping the raw sequence reads of N104 and P104 to the reference complete genome sequence of MAH 104 (NC_008595), we detected 27 SNPs. Of these, 19 SNPs were detected in both strains, 7 SNPs were specific to P104 and 1 SNP was specific to N104 (Fig. 3a). Using SnpEff to annotate the SNPs detected, we found that N104 had a nonsynonymous mutation that deleted the stop codon of *MAV_RS14660* (Table 3). Therefore, we focused on the SNPs detected only in the N104 strain that resulted in a fused protein consisting of two coding DNA sequences (CDSs), since the stop codon of *MAV_RS14660* is adjacent to the start codon of the CDS of *MAV_RS14655* (Fig. 3b, c).

**Table 3.** SNP information detected upon comparison with MAH 104 complete gene sequence (NC_008595)*

Strain	Position†	Nucleotide change	Amino acid change	Note
P104	9110	C>G	F630L	MAV_RS00035, DNA gyrase subunit A (c.1809C>G)
	34 551	C>T	P378L	MAV_RS00170, IS12445 family tranposase (c.1133C>T)
	119 380	C>G	P346R	MAV_RS00595, SuP family inorganic anion transporter (c.1037C>G)
	1 662 552	G>C	None	MAV_RS08090
	4 339 134	G>A	R117W	MAV_RS20195, DNA-binding response regulator (c.349C>T)
	4 620 124	C>G	K43N	MAV_RS21565, 30S ribosomal protein S12 (c.129G>C)
	5 186 500	G>A	None	MAV_RS24190
N104	3 139 242	T>C	†394W	MAV_RS14660, hypothetical protein (c.1181A>G)

*SNPs detected in both strains were excluded.

†Reference sequence (NC_008595) position where SNP was detected.

### N104 expressed a fusion protein of *MAV_RS14660* and *MAV_RS14655*

**Fig. 4.**
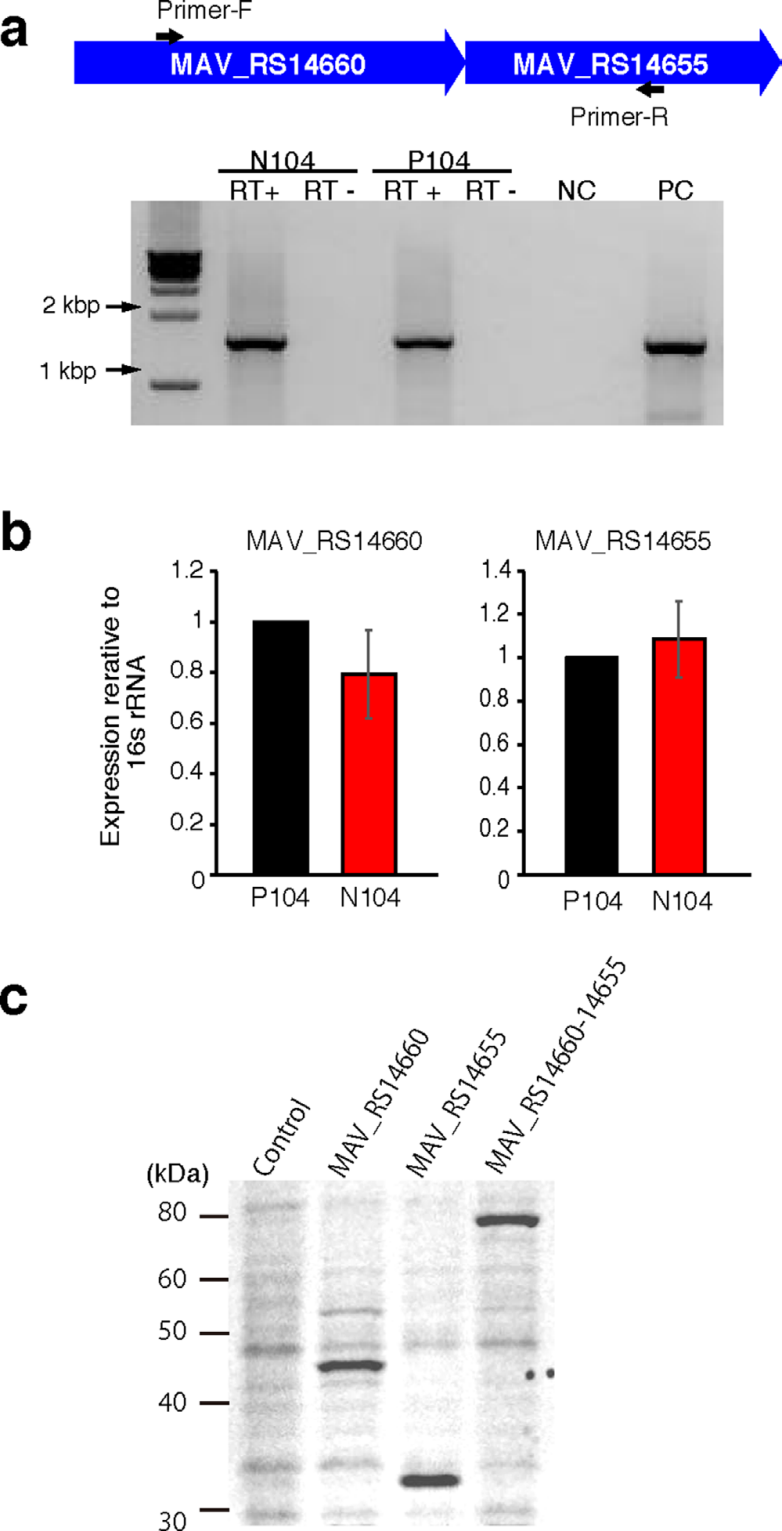
Analysis of mRNA and protein levels of *MAV_RS14660* and *MAV_RS14655*. (a) Schematic diagram of the primer set designed to amplify the region spanning *MAV_RS14660* to *MAV_RS14655*. The PCR amplicon was estimated at 1542 bp. Conventional reverse-transcription PCR was performed and the amplicons were subjected to agarose gel electrophoresis. Negative control, NC; positive control, PC. (b) Quantitative reverse-transcription PCR was performed and relative mRNA levels for each gene were calculated after normalizing to 16S rRNA levels. Data are represented as the mean±sd from four independent experiments. (c) Sodium dodecyl sulfate/polyacrylamide gel electrophoresis of recombinant MAV_RS14660 and MAV_RS14655 proteins and the fusion protein in the N104 strain. A recombined protein encoded by the fusion of sequences of *MAV_RS14660*, MAV_RS14655 (from P104) and *MAV_RS14660–14655* (from N104) followed by a poly-His-tag was expressed in *M. smegmatis *. The proteins in the bacterial pellets were detected based on the His-tag in the gel staining kit. Control, parent *M. smegmatis* mc^2^ 155 strain.

We used RT-PCR to detect the mRNAs for *MAV_RS14660* to *MAV_RS14655*, determine the presence of a fused mRNA (Fig. 4a), and quantify the gene expression of *MAV_RS14660* and *MAV_RS14655* (Fig. 4b). The mRNA levels for the two genes was similar in the N104 and P104 strains. Then, we investigated whether N104, which lost the stop codon between *MAV_RS14660* and *MAV_RS14655*, could express a fusion protein of these genes. Cloned nucleotide sequences of he *MAV_RS14660*, *MAV_RS14655* and *MAV_RS14660–14655* fusion sequence from the N104 strain were fused to the poly-His-tag sequence. These constructs were transformed into *M. smegmatis *. The size of the proteins encoded by *MAV_RS14660* and *MAV_RS14655* was predicted using Compute pI/Mw as 42 and 32 kDa, respectively. Recombinant proteins of the *MAV_RS14660–14655* fusion was detected at ~75 kDa using sodium dodecyl sulfate/polyacrylamide gel electrophoresis followed by His-Tag staining (Fig. 4c) Thus, this suggests that *MAV_RS14660* and *MAV_RS14655-*encoded proteins were expressed as one fusion protein in strain N104.

### An SNP was responsible for the deletion of the stop codon in strain N104

**Fig. 5.**
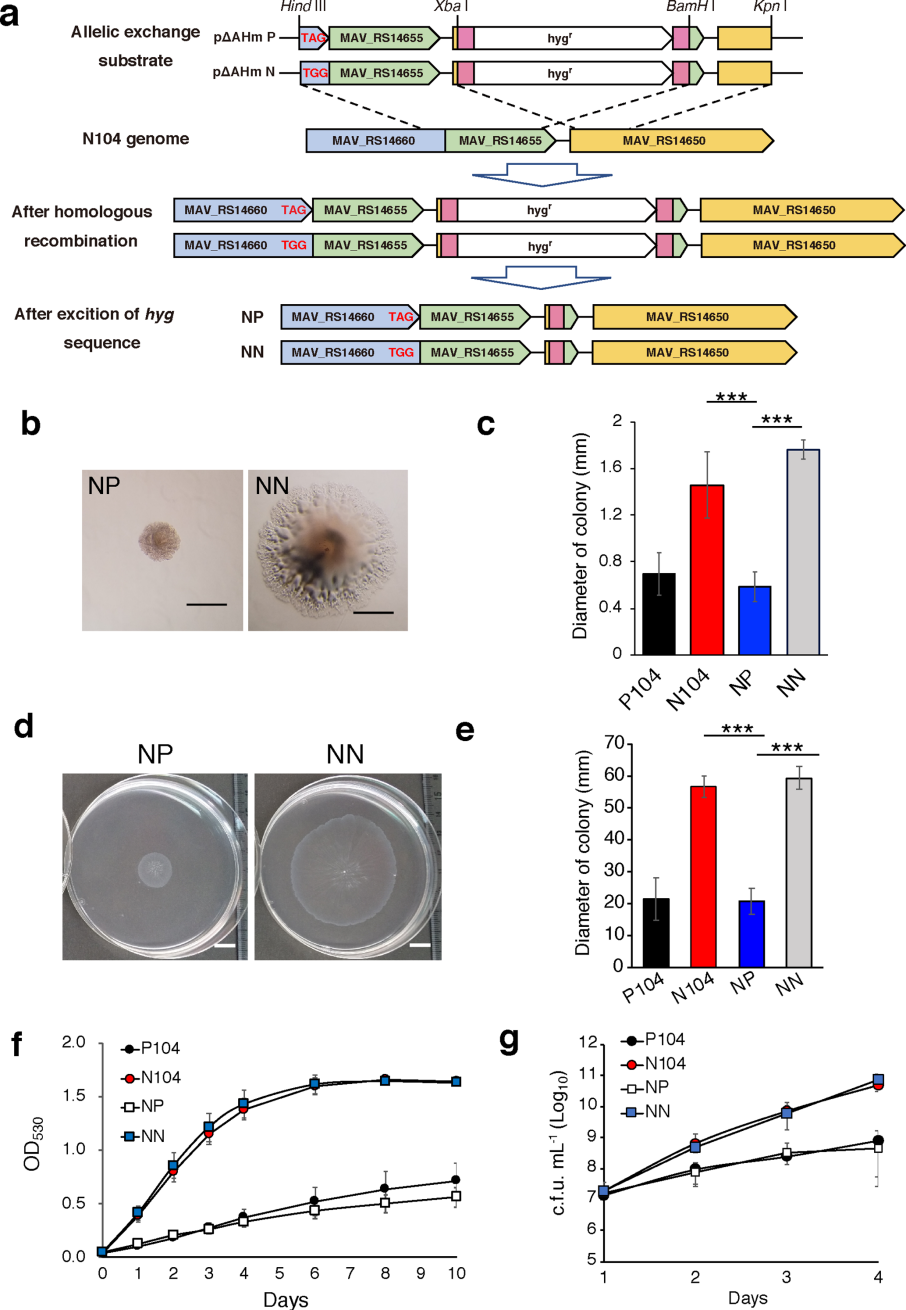
Assessment of the phenotype of N104-specific SNP using the revertant parent strain. (a) Schematic representing the introduction of the spanning region of *MAV_RS14660* and *MAV_RS14655* from the P104 genome into the N104 genome. The target codon for recombination is represented using red letters. Regions filled with pink represents res sites. (b) Representative images of colonies of MAH 104 strains on 7H10 agar after 8 days of growth. Left: revertant parent strain (NP). Right: strain with the N104 sequence into the N104 genome as control (NN). Scale bars represent 0.5 mm. (c) Diameter of colonies on agar were measured after *n* days of culturing, as indicated in the figure. Data are represented as the mean±sd of 20 samples in each group. (d, e) Motility of the MAH 104 strains on 0.3 % (w/v) agar plate. (f) Colonies on 0.3 % agar after 5 days of growth. Scale bars represent 1 cm. (g) Diameter of colonies were measured after 5 days. Data are represented as the mean±sd obtained from *n*=4. (f, g) Growth of the MAH 104 strains in 7H9 medium under conditions of shaking at 37 °C was assessed at OD_530_ (f), and c.f.u. (g) were enumerated after *n* days of culturing, as indicated in the figure. Data are represented as the mean±sd obtained from (f) *n*=5 and (g) *n*=4. Tukey’s HSD test was used for statistical analysis. ***P<0.005.

To ensure that the SNP in *MAV_RS14660* in the N104 strain is responsible for the phenotypes associated with strain N104, including rapid growth and high motility, we designed a revertant parent strain to replace the *MAV_RS14660* sequence of P104 to that containing the N104-specific mutation. The segmented sequence of the N104 genome containing the SNP was substituted with that of P104 containing the stop codon of *MAV_RS14660* using homologous recombination via the pJV53 vector (Fig. 5) [29]. The revertant strain contained the partial upstream sequence of *MAV_RS14660* from strain P104, the full-length sequence of *MAV_RS14655* from strain P104, and downstream portions of *MAV_RS14650* from strain P104. They were integrated into the ends of the res-hyg-res cassette, containing a gene encoding hygromycin resistance, arranged between the two δγ-res regions encoding the recognition sequence of resolvase. These DNA fragments were introduced into N104 transformed with pJV53. Eventually, the hygromycin resistance gene cassette was removed from the constructed revertant parent strain (NP) using δγ-resolvase. We constructed the control revertant strain, wherein the N104 mutation was replaced with that of the N104 sequence (NN), using the method described.

As expected, the NP strain produced significantly smaller colonies than those of the NN strain on solid agar (Fig. 5b), and the colony size was approximately the same as that of the P104 strain (Fig. 5c). Additionally, the spreading colony morphology of the NP strain on 0.3 % agar reduced to the same as that of the P104 strain (Fig. 5d, e). The growth rates of the NN and NP strains were assessed using liquid cultures with continuous shaking. The growth rate of the NP strain was significantly lower than that of the NN strain when analysed using OD_530_ and c.f.u. (Fig. 5f, g). These results demonstrated that the N104 phenotypes, including rapid growth and high motility, on solid agar were caused by an SNP in *MAV_RS14660*, resulting in the elimination of the stop codon.

### Functional assays of *MAV_RS14660* and *MAV_RS14655* gene products

**Fig. 6.**
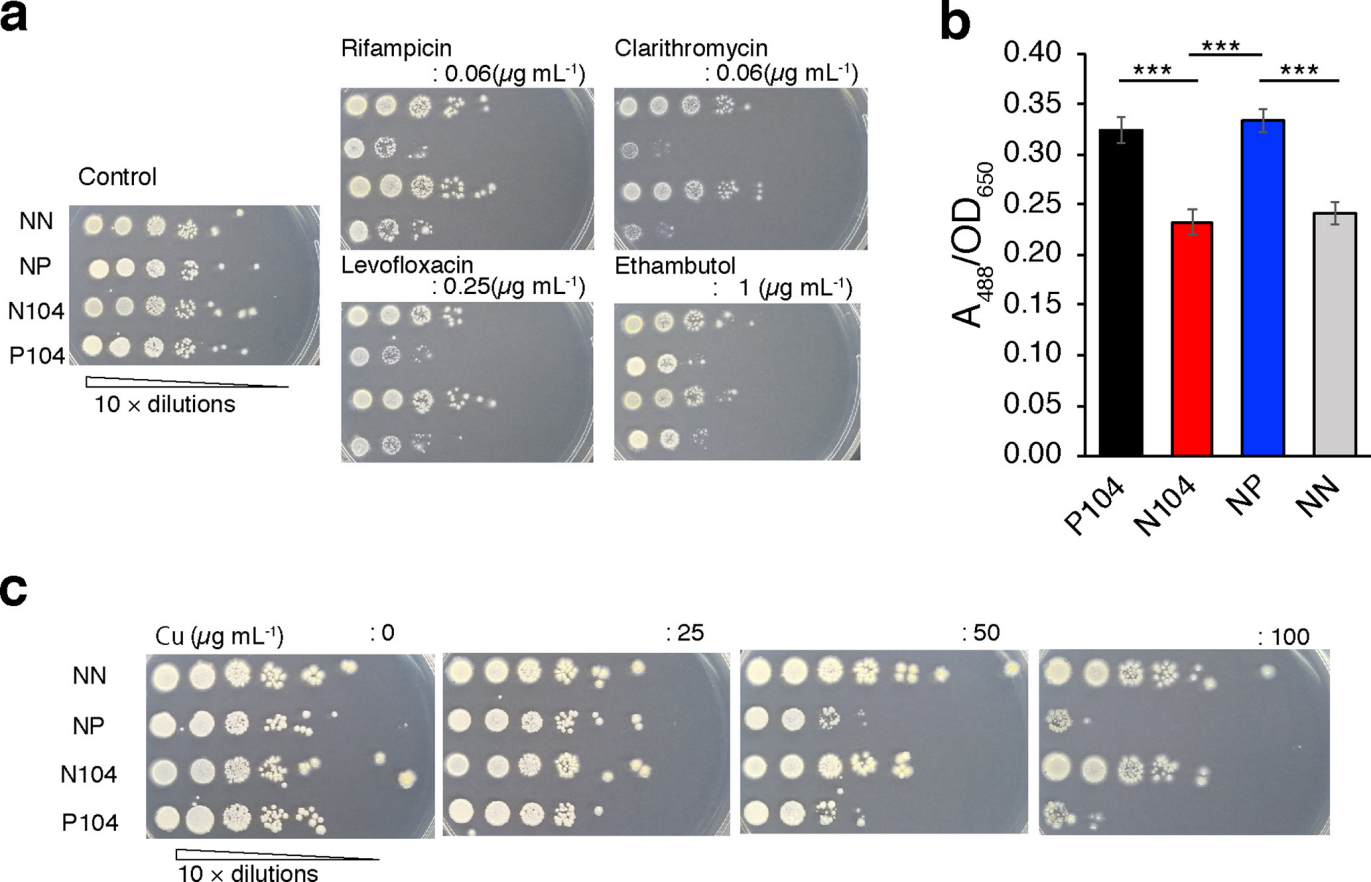
The effect of the mutation in the N104 strain on its antimicrobial susceptibility and copper resistance. (a) Susceptibility to antimicrobials was assessed by growing the cells for 15 days on 7H10 agar containing the following antimicrobial concentrations: 0 µg ml^−1^ (control plate), 0.06 µg ml^−1^ (rifampicin and clarithromycin), 0.25 µg ml^−1^ (levofloxacin) and 1 µg ml^−1^ (ethambutol). Inocula were prepared using 10-fold dilutions of cultures adjusted to 0.2 at OD_530_ and spotted (2 µl/spot). (b) The Congo red-binding ability of the P104, N104, NP and NN MAH 104 strains. Congo red binding was quantified by measuring *A*_488_ of Congo red divided by the OD_650_ of the bacterial cell suspensions. Data are represented as the mean±sd obtained from *n*=4. (c) Copper resistance was determined after growing the cells for 10 days on 7H10 agar containing 0, 25, 50, or 100 µM CuSO_4_. Inocula were prepared using 10-fold dilutions of cultures adjusted to 0.2 at OD_530_ and spotted (2 µl/spot). Tukey’s HSD test was used for statistical analysis. ***P<0.005.

Philalay *et al*. [32] reported that transposon-induced mutation in *MAV_RS14660* reduces the growth rate, attenuates antimicrobial resistance and enhances Congo red staining, indicating increased hydrophobicity [30]. Next, we tested the drug susceptibility and Congo red staining profiles of the MAH 104 strains. As shown in Fig. 6a, Table 4, the N104 and NN strains were more resistant to clarithromycin and rifampicin than the P104 and NP strains. The quantity of Congo red extracted from each strain demonstrated significant reduction in the uptake of Congo red by the N104 and NN strains compared to the P104 and NP strains (Fig. 6b).

**Table 4.** Antimicrobial susceptibility test of MAH 104 strains

Strain	Minimum inhibitory concentration (µg ml^−1^)			
	Clarithromycin	Rifampicin	Ethambutol	Levofloxacin
P104	0.06–0.125	0.03–0.06	1–2	0.125–0.25
N104	0.125–0.25	0.025	1–2	0.25–0.5
NP	0.06–0.125	0.06	1–2	0.25–0.5
NN	0.125–0.25	0.025	1–2	0.25–0.5

*MAV_RS14655* is the homologue of *Rv1698*, an outer-membrane channel protein in *Mycobacterium tuberculosis* that is involved in copper resistance by removing excess intracellular Cu ions [31]. Thus, we investigated whether the mutation leading to the fusion of *MAV_RS14660* and *MAV_RS14655* affected resistance to Cu in the MAH strains. The N104 and NN clones exhibited high resistance to 50 and 100 µM of CuSO_4_, while the parent clones failed to survive under these conditions (Fig. 6c). These data suggest that the mutation detected in the N104 strain might be related to the modified function of the *MAV_RS14660* and *MAV_RS1465* gene products.

## Discussion

In this study, we identified an SNP in the MAH 104 strain that replaced the stop codon of *MAV_RS14660* with tryptophan, which resulted in the fusion of *MAV_RS14660* and *MAV_RS14655*. Such gene fusions modulate bacterial phenotypes [33]. The fused gene in the MAH 104 strain was transcribed to produce a fused mRNA that was translated to form a fused protein, as predicted. Although the function of the MAV_RS14660 protein remains to be understood, Philalay *et al*. [32] reported that the transposon-mediated deletion of *MAV_RS14660* results in slower growth rates and enhanced Congo red staining, indicating increased susceptibility to multiple antimicrobials [34]. Our data demonstrated that the introduction of the SNP in *MAV_RS14660* in place of its stop codon in MAH 104 results in elevated resistance to specific antimycobacterials (Table 4, Fig. 6a) and decreased uptake of Congo red as compared with the capacity of the parent strain. These results suggest that the fusion of MAV_RS14660 and MAV_RS14655 proteins may be related to the enhancement in the function of the MAV_RS14660-encoding gene.

Colonies of the mutant N104 strain showed spreading colony morphology compared to that of the parent P104 strain (Fig. 1c, d). The presence or lack of GPLs, located on the outermost layer of the cell envelope, considerably influences important physiological processes, including sliding motility; the ability to translocate on the surface medium is related to the presence of GPLs [35, 36]. However, differences in the quantity of GPLs between N104 and its parent strain were not detected (Fig. S4). Colony size is influenced by sliding motility and growth rate. However, we were not able to exclude the possibility that the rapid growth of N104 was solely attributable to a rapid growth rate. It is also possible that the N104 strain exhibited enhanced sliding motility owing to increased spread colony phenotypes on solid medium at the stationary phase (Fig. S1). Moreover, the composition of lipids and lipoproteins in the outer membrane is presumably different because of differences in hydrophobicity, given the uptake of Congo red in the presence or absence of the stop codon in *MAV_RS14660* (Fig. 6a, b). The lipid components responsible for the motility and hydrophobicity of the N104 strain should be investigated in the future.

*MAV_RS14655* is located adjacent to the stop codon of *MAV_RS14660*. Rv1698, the homologue of MAV_RS14655 in *M. tuberculosis* (75 % homology in amino acid sequence), codes for a copper transport protein to protect mycobacteria from intracellular Cu ion toxicity. Therefore, we evaluated copper resistance between the mutants and parent strains. The N104 strain exhibited enhanced copper resistance (Fig. 6c), while the deletion mutant does not exhibit the same phenotype [31]. *Rv1698* codes for mycobacterial copper transport protein B (*mctB*) that is an outer-membrane protein and functions as a copper efflux channel [31, 37]. Copper resistance is essential for virulence in tuberculosis, since reduced Cu ion concentration contributes to the prolonged survival of mycobacteria within phagosomes in innate immune cells of the host [38]. However, it remains to be determined whether N104 survives longer within macrophages than the parent P104 strain.

The loss of a stop codon resulting in the production of a C-terminal extended protein is mechanically similar to translational readthrough by misreading [39] or stochastic frame-shift around a stop codon [40]. Unexpected elongation causes the protein to undergo misfolding and degradation. However, in some cases these errors lead to the formation of functional fusion proteins [33], which modify bacterial phenotype and/or enhance translation of other proteins [41, 42]. In this study, the strain had a tryptophan in place of the stop codon in *MAV_RS14660* that enabled the expression of the fusion protein without altering the transcription of *MAV_RS14660* or *MAV_RS14655*. Future studies should explore the molecular mechanisms by which the fusion protein directly or indirectly regulates cell functions and the localization of this fusion protein in the cell.

The molecular mechanism by which MAV_RS14660 protein functions remains to be understood. However, it may have an important role in mycobacterial growth, since its homologue is commonly conserved in mycobacteria. Fu *et al.* estimated that *Rv1697*, an orthologue of *MAV_RS14660* (90 % identity in amino acid sequence), plays a vital role in replication, translation and maintaining the growth rate of tuberculosis bacilli [43]. Interestingly, Goodsmith *et al*. showed that the membrane protein *PerM* is involved in magnesium-dependent cell division, and increased transcription of *Rv1697* and *Rv1698* was observed in *perM-*depleted *M. tuberculosis* [44]. MAV_RS14660 alone or in fusion with MAV_RS14655 may have similar roles in the acceleration of cell division. Notably, two fast-growing mycobacterial species, *M. smegmatis* and *M. abscessus *, exhibit the loss of function of *mctB* (*Rv1698/MAV_RS14655*) or express its homologous gene with low similarity, whereas these loci are well preserved in slow-growing *Mycobacterium* species. *Corynebacterium glutamicum* ATCC 13032 seems to have the same genetic organization (*recN/Rv1697(MAV_RS14660)/mctB/pyrG*) (accession no. NC_003450.3). However, the gene corresponding to *mctB* has low similarity and all of their functions are unknown. Thus, it seems worthwhile to investigate if the activity of these loci affects growth in mycobateria.

In conclusion, we have identified a substitution mutation that replaces the stop codon with the tryptophan-encoding codon in *MAV_RS14660* that is responsible for the increased growth rate and spreading colony morphology in the slow-growing MAH 104 strain. Future experiments should focus on investigating the underlying molecular mechanisms for the enhanced growth to advance our knowledge base concerning the biology of *Mycobacterium* species and issues of clinical significance, such as antimicrobial drug resistance.

## Supplementary Data

Supplementary material 1
