# Supplementary material for: Point mutation in the stop codon of MAV_RS14660 increases the growth rate of Mycobacterium avium subspecies hominissuis
Source: Microbiology (Reading). 2020 Dec 23;167(2):001007. doi: 10.1099/mic.0.001007 (PMC8131024; doi:10.1099/mic.0.001007)
Supplement: Supplementary material 1 [file mic-167-007-s001.pdf]

Supplemental methods

Measurement of diameter of colonies

Colony morphology was observed after 21 days of culture on 7H10 agar. The diameter of the colonies (major axis) was measured from images.

Analysis of bacterial growth

The initial bacterial suspension was adjusted to 0.04–0.05 OD_{530} and 10 mL of this suspension was incubated at 37°C with shaking. Bacteria were cultured until the OD was saturated and wet volume of 5 mL of culture was measured as per the method reported by Elguezabal N et. al.[1]. Briefly, 5 mL of culture was transferred to a weighted 15 mL conical tube and centrifuged at 2,380 ×*g* for 10 min. The supernatant was discarded, and residual water removed by inverting the tube upside down for 15 min. Subsequently, the tube was weighted, and the wet weight was calculated by subtracting the weight of the empty tube from the weight of the tube and pellet.

Electron microscopy and measurement of bacterial size

Morphological analysis of bacterial cells using scanning electron microscopy (SEM: IT-300, JEOL, Tokyo, Japan) was performed as described previously[2]. Briefly, colonies on 7H10 agar for more than 20 days culture were fixed with 2.5% (w/v) glutaraldehyde in 0.1 M phosphate buffer (PB; pH 7.4) at 4°C overnight and rinsed thrice with PB. Next, the samples were fixed with 1% (w/v) osmium tetroxide for 1 h at 4°C and dehydrated using graded series of ethanol. After substituting with *t*-butyl alcohol, the samples were dried using a freeze drying device (JFD-320, JEOL, Tokyo, Japan) and coated with gold using an ion spatter coater (Q150 ES Plus, Quorum Technologies, Laughton, UK). The samples were observed using SEM. The short axis of bacteria was measured from the acquired electron micrographs. Morphological analysis of bacterial cells using cryo-transmission electron microscopy (Cryo-TEM) was performed as described previously[3]. Briefly, 7H9 bacterial cultures were grown to ~0.5 OD_{530} under conditions of shaking and were fixed with 2.5% (w/v) glutaraldehyde in PB at 4°C overnight. Then, after centrifugation at 10,000 × g, the supernatants were discarded and pellet was rinsed with PB. Finally, the pellets were resuspended in 200 μL of PB from which 1 μL of suspension was applied to a glow-discharged carbon grid with holes (Quantifoil copper grids R 2/1 or S 7/2, Quantifoil MicroTools, Jena, Germany) and mounted in an environmentally controlled chamber at 100% humidity. The grids were frozen in vitreous ice by plunging them into a liquid ethane-propane mixture cooled with liquid nitrogen using EM GP2 (Leica Mikrosysteme GmbH, Vienna, Austria). The grid was loaded in Single Tilt Liquid Nitrogen Cryo Transfer holder (Model 626, Gatan Inc., Pleasanton, California) and subjected to TEM (JEM-1230, JEOL, Tokyo, Japan). The microscope was

operated at 120 kV acceleration voltage and, during examination, the samples on the grids were cooled with liquid nitrogen to a temperature of -175.9–-177.8°C. Raw images of the intact cells were recorded at magnifications of ×10,000, ×15,000, or ×20,000 according to the length of a single cell using the 1K × 1K CCD digital camera system (OSIS MegaView G2, Olympus, Tokyo, Japan). The images captured with Cryo-TEM were analyzed using the Measure command in the Analyze menu of ImageJ/Fiji[4].

Thin layer chromatography (TLC) analysis of glycopeptidolipids (GPLs)

Isolation and analysis of GPLs were performed as described previously [5]. Briefly, the bacterial cells were harvested from 2 – 3 weeks cultures on 7H10 agar and their total lipids were extracted with $CHCl_3/CH_3OH$ (2:1, v/v). GPLs were purified from total lipids using alkaline hydrolysis and spotted on TLC plate (Merck, silica gel 60 F_{254}). TLC was performed using $CHCl_3/CH_3OH$ (9:1, v/v) solvent and visualized by spraying with a solution of 10% H_2SO_4 and heating.

Supplemental references:

1. **Elguezabal N, Bastida F, Sevilla IA, González N, Molina E, *et al.*** Estimation of Mycobacterium avium subsp. paratuberculosis growth parameters: Strain characterization and comparison of methods. *Appl Environ Microbiol* 2011;77:8615–8624. doi: 10.1128/AEM.05818-11.
2. **Yamada H, Yamaguchi M, Igarashi Y, Chikamatsu K, Aono A, *et al.*** Mycolicibacterium smegmatis, Basonym Mycobacterium smegmatis, Expresses Morphological Phenotypes Much More Similar to Escherichia coli Than Mycobacterium tuberculosis in Quantitative Structome Analysis and CryoTEM Examination. *Front Microbiol* 2018;9:1992. doi: 10.3389/fmicb.2018.01992.
3. **Yamada H, Bhatt A, Danev R, Fujiwara N, Maeda S, *et al.*** Non-acid-fastness in Mycobacterium tuberculosis ΔkasB mutant correlates with the cell envelope electron density. *Tuberculosis* 2012;92:351–357. doi: 10.1016/j.tube.2012.02.006.
4. **Schindelin J, Arganda-Carreras I, Frise E, Kaynig V, Longair M, *et al.*** Fiji: An open-source platform for biological-image analysis. *Nat Methods* 2012;9:676–682.
5. **Miyamoto Y, Mukai T, Takeshita F, Nakata N, Maeda Y, *et al.*** Aggregation of mycobacteria caused by disruption of fibronectin-attachment protein-encoding gene. *FEMS Microbiol Lett* 2004;236:227–234. doi: 10.1016/j.femsle.2004.05.047.

FIG. S1.

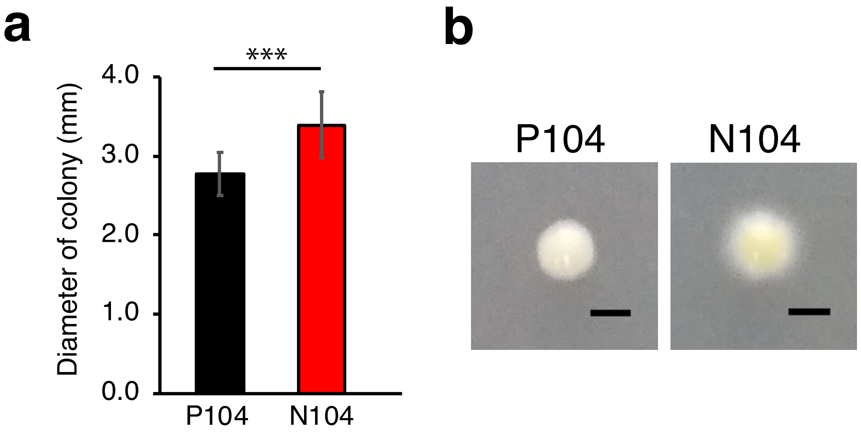

Colonies of P104 and N104 after 21 days of culturing. (a) Diameter of colonies on 7H10 agar were measured using 21-day cultures. Data were represented as mean±standard deviation (S.D.) of 23 samples in each group. (b) Representative images of colonies from the two strains on 7H10 agar after 21 days of growth. Scale bars represent 2 mm. Student's *t*-test was used for statistical analysis. ***P<0.005.

FIG. S2.

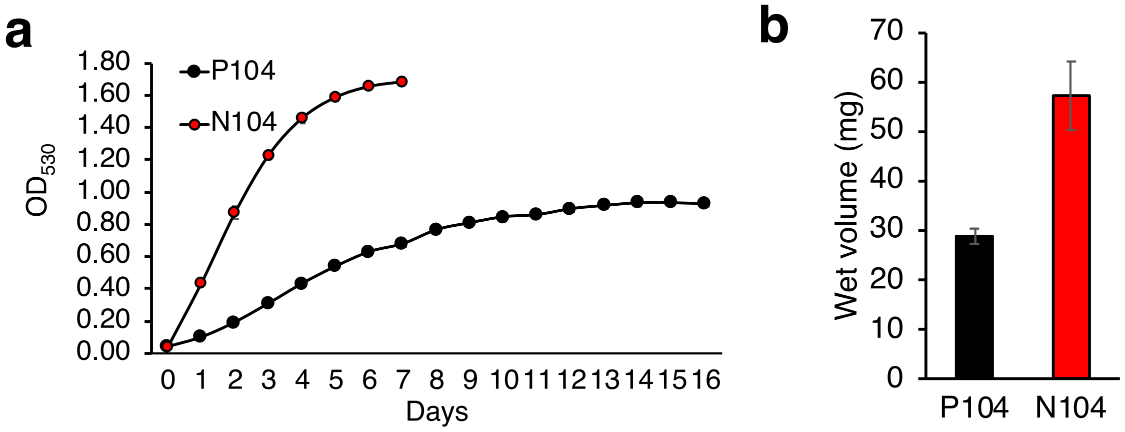

Analysis of bacterial wet growth. (a) Growth of MAH 104 strains in 7H9 medium under conditions of shaking at 37 °C and OD_{530}. Data was represented as mean ±S.D. obtained from n=3. (b) Wet volume of bacterial cells was measured from cultures harvested at stationary phase (after 16 days of culture for P104, and after 7 days of culture for N104). Data was represented as mean ±S.D. obtained from n=3.

FIG. S3.

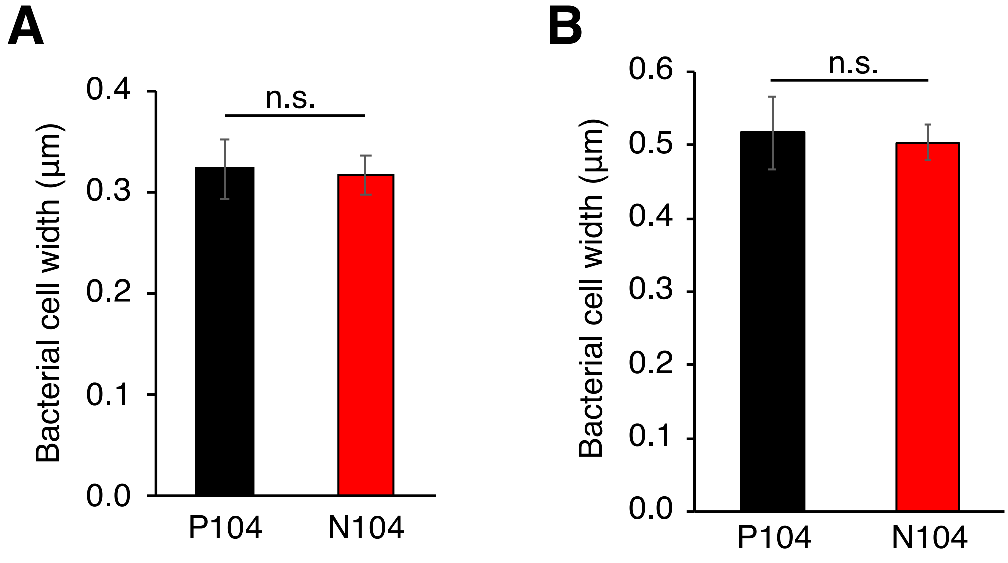

Bacterial cell length measured using scanning electron microscopy and cryo-transmission electron microscopy. (a) Width of bacterial cells cultured on 7H10 agar was measured from scanning electron micrographs. Data were represented as mean ±S.D. obtained from 50 samples in each group. (b) Width of bacterial cells cultured in 7H9 broth was measured from cryo-transmission electron micrographs. Data were represented as mean ±S.D. obtained from 33 samples of P104 and 43 samples of N104. Student's *t*-test was used for statistical analysis. n.s.; not significant.

.

Fig. S4

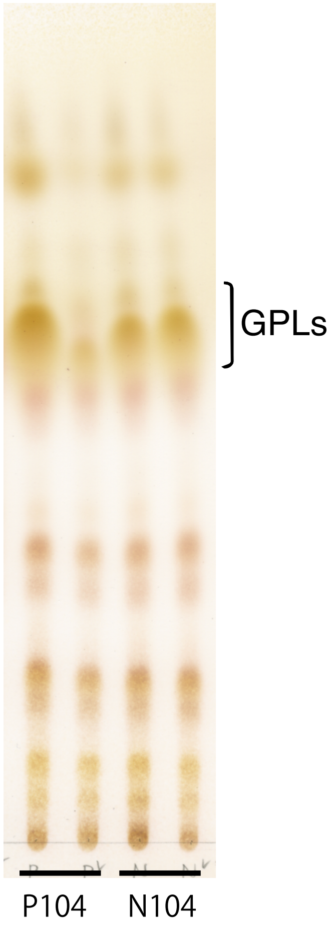

TLC analysis of glycopeptidolipids (GPLs) extracted from strains MAH P104 and N104. GPLs were purified from total lipids using alkaline hydrolysis. GPLs of MAH P104 and N104 were spotted on silica plate and developed using $CHCl_3$-CH_3OH (9:1 [vol/vol]).
